# Sustainable production of healthy, affordable food in the UK: The pros and cons of plasticulture

**DOI:** 10.1002/fes3.404

**Published:** 2022-07-06

**Authors:** Samuel J. Cusworth, William J. Davies, Martin R. McAinsh, Carly J. Stevens

**Affiliations:** ^1^ Lancaster Environment Centre Lancaster UK

**Keywords:** crop production, food security, growing systems, plastic pollution, plasticulture, protected cropping

## Abstract

An evolving green agenda as the UK seeks to achieve ‘net zero’ in greenhouse gas emissions by 2050, coupled with our new trading relationship with the European Union, is resulting in new government policies, which will be disruptive to Britain's traditional food and farming practices. These policies encourage sustainable farming and land‐sparing to restore natural habitats and will provide an opportunity to address issues such as high emissions of GHGs and dwindling biodiversity resulting from many intensive agricultural practices. To address these and other food challenges such as global conflicts and health issues, Britain will need a revolution in its food system. The aim of this paper is to make the case for such a food revolution where additional healthy food for the UK population is produced in‐country in specialised production units for fruits and vegetables developed on sites previously considered unsuitable for crop production. High crop productivity can be achieved in low‐cost controlled environments, making extensive use of novel crop science and modern controlled‐environment technology. Such systems must be operated with very limited environmental impact. In recent years, growth in the application of plasticulture in UK horticulture has driven some increases in crop yield, quality and value. However, the environmental cost of plastic production and plastic pollution is regarded as a generational challenge that faces the earth system complex. The distribution of plastic waste is ubiquitous, with a significant pollution load arising from a range of agricultural practices. The primary receptor of agriplastic pollution is agricultural soil. Impacts of microplastics on crop productivity and quality and also on human health are only now being investigated. This paper explores the possibility that we can mitigate the adverse environmental effects of agriplastics and thereby exploit the potential of plasticulture to enhance the productivity and positive health impact of UK horticulture.

## Graphical abstract

This paper is submitted to the FES Special Issue on Food Security. It focusses on two current issues, namely the need for a food system revolution for the UK and the possibility that UK can become more self‐sufficient in food. The increased production of healthy food (more fruit and vegetables) is particularly important. Possibilities for new food production systems are discussed and there is some focus on the topical issue of plastic pollution with particular reference to methods of protected cropping. The accumulation of microplastics and nanoplastics in agricultural soil is currently causing some environmental and health concerns. In the light of current global challenges the resilience of our food system is important.

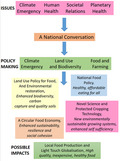

## 1 INTRODUCTION: CURRENT CHALLENGES FOR THE UK FOOD SYSTEM

Global food security can be defined as a situation where all people at all times have physical, social and economic access to sufficient safe, nutritious and sustainably‐produced food that meets their dietary needs and preferences (Global Food Security, 2021). There is now increasing confidence that within the next 25 years our society will have the capability, the resources, the knowledge and the technology to feed an expanded global population while delivering improved human‐ and planetary health (Global Food Security, 2021; Horton et al., 2021).

Despite this confidence, we are still faced with a necessity to address a range of significant food‐related challenges. Perhaps foremost among these is the development and the impact of the climate emergency. A recent report from the Intergovernmental Panel on Climate Change (IPCC, 2021) highlights with high confidence the observation that elevated temperatures and increased drought are already significantly negatively impacting agriculture, food availability, food access, food utilisation and food stability on most continents of the world. At the same time, our global food system currently contributes a third of global greenhouse gas (GHG) emissions, thereby driving further climate change (Crippa et al., 2021) and this issue must be addressed with some urgency. GHG emissions from grazing ruminants and accompanying feed production, from soil tillage and from the significant production of the rice crop in several parts of the world are identified as significant targets for those seeking to increase the sustainability of food and farming. Overuse of input resources such as water, nutrients and other agrochemicals coupled with the use of some inappropriate farming practices are also environmentally damaging (e.g. Willett et al., 2019). In the UK there is some regulation of water use, yet there is still scope to improve the water productivity of many crops (e.g. Morison et al., 2008).

Deteriorating public health is a growing challenge in many regions of the world and malnutrition is now a leading cause of early deaths globally (Global Food Security, 2021). More than half of the global population is now underweight, overweight or obese (FAO, 2021). By 2035, the UK National Health Service is predicted to be spending more on complications of type 2 diabetes (often a consequence of a poor diet) than its entire spend on cancer treatment (Royal Society of Biology, 2021) and two‐thirds of the UK population may be malnourished. The UK's childhood obesity problem is particularly acute (Gov UK, 2020; World Health Organisation, 2020). While addressing these problems requires us to take account of many socio‐economic challenges and modify much consumer behaviour, we must also work to improve people's access to healthy and sustainable foods (Willett et al., 2019).

Making more healthy food available to people in the UK is a significant challenge as the UK cost of living rises and could require an increased focus on food produced in the UK by organisations ranging in scale from multi‐national suppliers to local producers. Our focus must also be on more reliable international trade, as shown by a recent report supported by the UK food retailer Morrisons (Benton et al., 2017). This report shows that while the UK produces 52% of the food that we eat in the UK, only 23% of the fruit and vegetables consumed are produced here, (percentages based on the farm gate value of the crops). The EAT Lancet commission and others (Benton, 2019; Willett et al., 2019) emphasise the importance of increasing the proportion of fruit and vegetables in diets, particularly those of young people. There is general recognition that reduced consumption of meat in most diets in the developed world would have both dietary and environmental benefits.

And generating environmental problems from our own agricultural activities (e.g. GHG emissions and other environmental issues such as overuse of water and fertiliser), we import food from nearly 200 countries across the globe (Benton et al., 2017), thereby generating emissions and other pollution and resource use issues offshore as a result of both production and transport of food. Any changes in our food system driven by dietary requirements must take into account the impact of such changes on planetary health. The UK has committed to achieving ‘Net Zero’ by 2050 (Gov UK, 2021), and this will likely require significant changes to environmental accounting both at home and abroad. Despite our requirements for more overseas trading partners, it will be hard to sign new trade deals with countries with lower food production emissions and health standards than our own. This challenge has been compounded by procedural and political changes post‐Brexit. Recently published proposals for a National UK Food Strategy (UK National Food Strategy, 2021) highlights many of these issues in some detail. The strategy aims to deliver healthy, affordable and safe food for all in the UK. Emphasis is placed on increasing the resilience and sustainability of food production and supply but also on restoring and enhancing our natural environment.

In the UK, in recent years some discussion has focussed on rewarding farmers for good environmental stewardship, rather than for productivity. The Government's new Environmental Land Management (ELM) strategy is now being set out (January 2022). This is expected to lead to significant changes in land management, benefiting the natural environment and addressing the challenges of the climate emergency. ELM will pay farmers for undertaking actions to improve the environment. It has three components, each of which will be launched in full in 2024 (NAO, 2021):

- The Sustainable Farming Incentive (SFI) will be open to all farmers and will pay them for actions to manage their land in an environmentally sustainable way.
- Local Nature Recovery will pay for more complex actions that deliver benefits at a local level and aims to encourage collaboration between farmers.
- *Landscape Recovery will support large‐scale projects to deliver landscape and ecosystem* Although there is much concern across the farming community about the withdrawal of much financial support currently available to reward farmers for food production there is general recognition that the UK must give increased attention to maintaining and even enhancing biodiversity, as well‐functioning ecosystems are critical for human existence, economic prosperity and a good quality of life. A healthy, diverse biosphere aids in the provision of food, energy, shelter and medicines. It will also sustain water and soil quality and help regulate the Earth's climate. Early ELM proposals released in January 2022 have caused some alarm among the UK farming community. There are reservations about the rewards that will be available to small farmers (compared with the current support arrangements) *recovery through long‐term land‐use change projects such as large‐scale tree planting and peatland restoration*.

and worries that current proposals cannot reward tenant farmers who currently make up almost half of UK farmers (13% of farm holdings are wholly tenanted and 34% are ‘mixed tenure’, DEFRA, 2019). Small scale farmers may circumvent these barriers by forming a collective group to pass the land area threshold but are then met with other challenges that come with collaboration (DEFRA, 2020).

The recent National Biodiversity Network (NBN) report on biodiversity in the UK (NBN, 2021) shows that we retain only half of our natural biodiversity, a disturbing figure, especially when compared to other G7 countries. The UK is at the very bottom of the G7 table in terms of how much biodiversity still survives and is in the bottom 10% of all countries globally. Loss of soil biodiversity may be particularly critical as changes in soil communities and the loss of biodiversity threaten ecosystem multifunctionality and sustainability (Wagg et al., 2014). There are around 11 million species of soil organisms, but fewer than 2% have been named and classified and a 2019 Environment Agency report indicated that the UK soil invertebrate community has not been fully surveyed since 2007. This highlights the evidence gap for one of the major indicators of soil health. Results from this survey indicate significantly fewer invertebrates in arable habitats than in other habitats (Emmett et al., 2010). There are considerable data showing worldwide decline in soil health and soil biodiversity (e.g. FAO et al., 2020).

The Intergovernmental Science‐Policy Platform on Biodiversity and Ecosystem Services (IPBES) has reported that land‐use changes have had the greatest overall negative impact on nature since 1970 (IPBES, 2019). Unsustainable agriculture, logging, transport infrastructure, residential and commercial development, energy production and mining are all common problems in this context. Where and how food is produced has been one of the biggest drivers of land‐use change (World Wildlife Fund, 2020). This issue is acute in the UK where 70% of our land area is devoted to agriculture, this is around 17 million hectares, of which 6 million hectares are under cultivation for cereals, oilseeds, potatoes, salads, fruit and vegetables, with the remaining land used for grazing and raising livestock (Benton et al., 2017). A dietary change involving reduced meat consumption to address health and environmental issues potentially frees up land previously used for grazing and production of animal feed and opens the possibility for the development of new agricultural systems to allow for enhanced fruit and vegetable production. Such a dietary change also brings benefits in terms of reduced CO_2_ emissions and nutrient losses (Foly, 2022).

Consideration of this welter of new policies on food and farming does suggest a possible conflict between policies designed to encourage the production of more food in the UK and large‐scale projects to deliver landscape and ecosystem recovery through changes in long‐term land‐use (such as large‐scale tree planting and peatland restoration for carbon capture and storage). More ‘extensive’ agriculture, will almost by definition reduce the production of some foods. If enough land is to be spared to store enough carbon to offset emissions from UK farming, then a proportion of what remains dedicated to food production will have to focus on intensive production (Balmford, 2021). Such intensification is potentially problematic for the welfare of animals, but advances in modern plant biology and the exploitation of novel engineering solutions may provide exciting opportunities for agronomy and horticulture in the UK (UKPSF, 2019).

## 2 SCENARIO PLANNING: HOW DO WE ADAPT OUR FOOD SYSTEM FOR THE FUTURE IN ORDER TO COPE WITH GROWING PRESSURES ON UK SOCIETY?

The UK Global Food Security (GFS) programme (Global Food Security, 2021) has addressed this and related questions implicit in our efforts to define and develop a global food system that is better at feeding us while it is also better at looking after the planet. They have done this by scenario planning, which considers different UK responses to two much‐lauded, landmark global agreements: the Paris Agreement on climate change and the UN's Sustainable Development Goals (SDGs). Importantly, ‘*the scenarios in this report do not aim to predict what will happen in the future, nor do they suggest what the preferred future might be. They are designed to stimulate thought, identify opportunities and threats that the UK food system may face in the future, and aid long‐term decision‐making’*. (Global Food Security, 2021).

In the GFS exercise, scenarios were developed based on two critical uncertainties that are expected to drive changes to the UK food system in the coming years. One uncertainty was whether by 2050 the UK food system would rely more on local production and supply or upon greater globalisation to make food available to the UK population? The second uncertainty was a consideration, over the same time period, of what would be the impact on the UK food system if it were transformed to deliver either some climate mitigation (as suggested in the Paris Agreements) or to deliver on a broader range of sustainability metrics (i.e. the issues implicit in nearly all of the Sustainable Development Goals (SDGs)).

**FIGURE 1:**
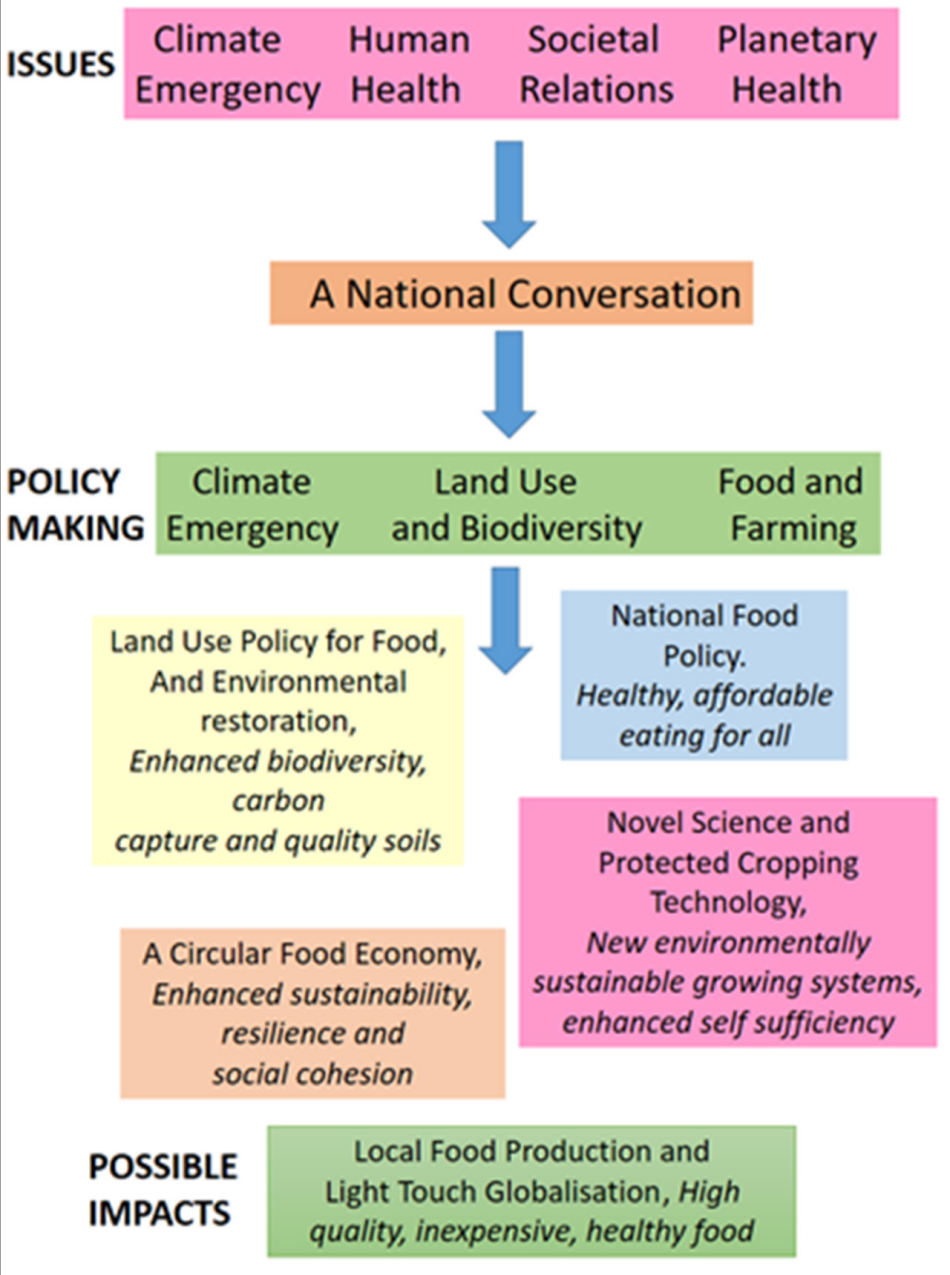
A localised communal food system developed in the UK in response current environmental and social issues. Impacts are developed within a framework of possibilities suggested by UK Plant Sciences Federation (2019) and Global Food Security (2021)

In all four scenarios (localised system focussed on general sustainability targets, localised /climate mitigation targets and two globalised scenarios with climate or general sustainability foci) developed by the GFS exercise, the proposal is that climate change will contribute directly or indirectly to higher costs in the food system. Another major conclusion is that, as suggested in other recent papers (e.g. Willett et al., 2019), the elimination of food waste in both the food supply chain and in the household combined with a shift towards diets with reduced animal protein is highly likely to be of major importance in the delivery on the respective global agreements. A recent report by the Copernicus Climate Change Service (Copernicus, 2022), highlights the potential rate of future climate change and they note that the last seven years have been the hottest on record for the planet. GFS scenario planning and our own historical experience of the development of the global food system suggest that a focus only on reducing the effects of climate change on the operation of the food system in the UK could undermine key issues for society, such as biodiversity, human health and the economic well‐being of many people. Nevertheless, the GFS exercise also suggested that both greater self‐sufficiency in food and multilateral cooperation could to some degree protect the UK food system against future climate disruption of food supply. Because of the current opportunities offered to the UK by political changes affecting food and farming policy we now consider how a more‐localised, communal UK food system might be developed and how it might help deliver benefits to both human and planetary health (see Figure 1).

Among several scenarios from the GFS, exercise is the possibility that the national food system could be characterised by ‘a local‐production for‐local‐consumption ethos’. Local crop production facilities might allow high yields of reasonably priced, healthy food in more months of the year. Such changes, stemming from novel protected cropping might allow some increase in UK food self‐sufficiency. Novel production facilities might be located in urban and peri‐urban locations, potentially reducing the demand for agricultural development in rural locations (Walsh et al., 2022). This demand might be further reduced by changes in diet and reduced demand for animal protein thereby allowing land sparing for environmental restoration, carbon capture from reforestation and some green energy production. In making changes to land use of this kind, care should be taken to ensure that habitats of high conservation value are not lost in a change to more productive habitat types.

The GFS Food Scenarios project further foresees that UK agricultural policy, currently under development, supports the production of climate‐friendly nutritious foods. Future health policy might use financial incentives to encourage citizens to adopt healthy, environmentally sustainable diets, although the 2022 food price challenges have delayed proposed policy changes in this area. The hope is that the typical UK diet will include more fruit and vegetables, and less sugar, starch and fat. One particularly significant GFS suggestion is that as a result of likely agricultural diversification, UK food production could be made more resilient to changing weather patterns resulting from predicted climate change. We now consider the possible evolution of the UK food system in more detail.

## 3 AN EVOLVING UK FOOD SYSTEM

In this section, we consider the question of whether it is possible for the UK to increase the quantity and diversity of fruit and vegetables grown in the UK. This requires a brief look at some recent historical development of our food production systems.

The 18th and 19th centuries saw the development of glasshouses for food production in the UK. Food storage technologies also improved during this period and increased use of pest and disease control measures for both crops in the field and in storage also increased food availability. The development of plant and animal genetics has further increased yields with agronomical advances contributing to reduced yield gaps (the difference between potential and actual crop yield). Over the years the practice of highly‐productive intensive agriculture with high input resources has evolved. In combination with a highly efficient import/export trade, this has resulted in the development of a sophisticated supply chain for food for the British public. Diets of many are largely determined by costs of food, but for the more affluent, the policies of retailers have been devoted to supplying whatever customers demand whenever they want it, whatever the monetary and environmental cost. This situation is likely to change as the Government legislates to make more healthy food, produced in an environmentally‐friendly fashion, available to more people. However, while Britain's food and farming policy has resulted in an enviable productivity and an efficient supply chain, intensive farming practices are now commonly blamed for Britain's biodiversity crisis and a significant portion of the UK's GHG emissions. Activists and policymakers are beginning to imagine new types of food production for the UK, which are more planetary friendly.

Britain's colonial history has made available a broad range of exotic crops from different parts of the world as food sources for the British population. Lang (2020) has noted that the historical strength of the British Navy has ensured the security of these supply lines. However, global conflict and a general reduction in Britain's global influence have increased the potential fragility of the UK's supply chains (Lang, 2020). Supply chain instability may require more domestic production to ensure greater food security for the UK population (and a reduction in the environmental footprint of the food items). It is important therefore to consider the degree to which this policy conflicts with the UK Government's land‐use policies where farmers are rewarded primarily for the delivery of public goods with seemingly little encouragement to produce more, good quality food.

Many of the fruits (and some of the vegetables) that are consumed in the UK are intolerant of freezing‐ or even chilling temperatures. Some countries with climates colder than the UK do grow commercial quantities of exotic fruits and vegetables under protected cropping (e.g. bananas in Iceland), but the likelihood of being able to grow significant quantities of such ‘exotics’ for mass consumption in the UK would be prohibitively expensive, both financially and environmentally.

Suppliers of fruit and vegetables to the UK market have demonstrated best practices in growing staple crops for the UK in overseas environments where radiation levels and temperatures are high enough for very high productivity (e.g. G's Fresh (2022) and Barfoots (2022)). The sustainability of such operations has in the past been questioned because of the ‘food miles’ involved, but these farming operations in many countries where the climate delivers high productivity can supply the UK market with a product with a lower total global warming potential (GWP) than is the case for UK production (e.g. Webb et al., 2013). This will only be the case if refrigeration and transport costs are low (as is the case in the shipping of vegetables from West Africa by sea. In this case, transportation to the UK takes only 4–5 days and so this highly‐productive year‐round production system has to this point in time (2021) fitted well with the ‘just‐in‐time’ supply chains used by most UK retailers. Whether this will continue to be the case as the consequences of Brexit unwind, remains to be seen. A future UK food policy that is climate‐friendly and supports the production of nutritious foods is likely to involve imported food grown and transported appropriately to the UK market. Nevertheless, as suggested by GFS (2021), the possibility that the national food system could be characterised by ‘a local‐production for‐local‐consumption ethos’ deserves serious consideration. We must therefore ask how this can be achieved. The urgency of providing answers to this question is emphasised by the current (May 2022) conflict in Ukraine. This has involved two countries that contribute significant amounts of food to the global food system and we are already experiencing changes in both the availability and the cost of food. A recent report from Chatham House (Benton et al., 2022) proposes that governments must invest now to build the long‐term resilience of societies and economies against global shocks such as Russia's war in Ukraine and the COVID‐19 pandemic. The Chatham House report emphasises that effects of serious market disruption and geopolitical upheaval should be buffered by what the authors’ term ‘no‐regrets’ measures. As we have already emphasised, a national food system with extended food supply chains is highly vulnerable to disruption. Enhanced UK food production will help to buffer the effects of external shocks to our food system and might be thought of as such a ‘no‐regrets’ measure. Introduction of measures of this kind will require a national debate with, among others, the general public. These considerations are further discussed later in the paper.

**FIGURE 2:**
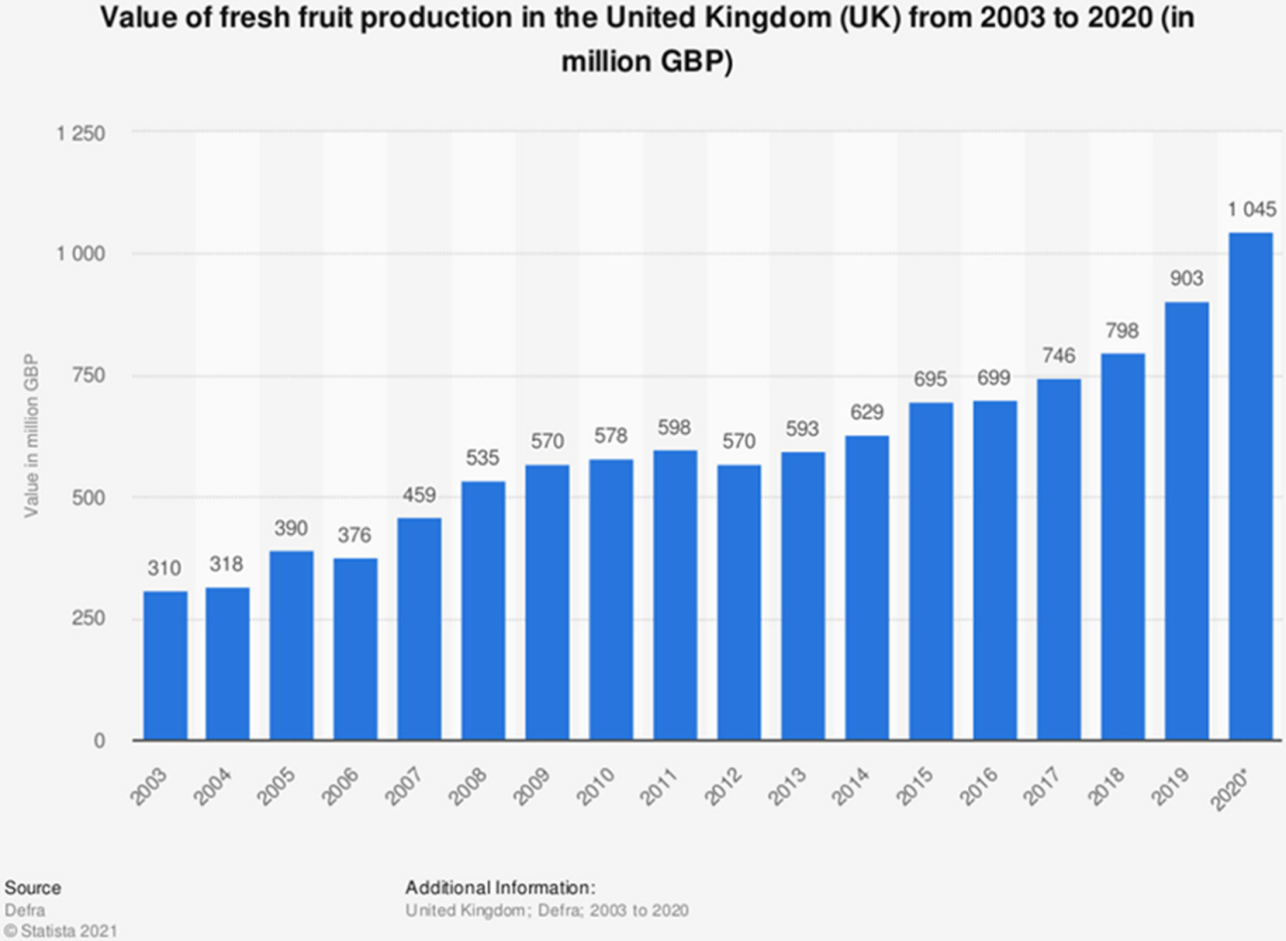
Cusworth et al. Data from GOV.UK and DEFRA (2021) illustrate a general increase in the value of fresh fruit produced within the UK from 2003 to 2020 (in million GBP)

In recent years, horticultural production has been a success story for the UK (DEFRA, 2021). The value of home‐produced vegetables increased by 10% to just under £1.7 billion in 2020, and the volume of home vegetable production also increased in the year 2020 by 3%, compared with the year 2019. Home‐produced fruit has grown in value to £1.0 billion, an increase in 16% compared with 2019 (see Figure 2), with production volumes falling 4.5% (DEFRA, 2021). We now examine the basis of these changes and ask whether further gains are possible using technology and genetic material accessible to the industry.

There are many reasons for increases in crop productivity and value shown over the last several years, but prominent among these is the availability of new varieties generated by breeding programmes for many crops, which have been successful in generating enhanced fruit and vegetable quality and yield, and some degrees of stress tolerance (e.g. https://www.harper‐adams.ac.uk/research/project/148/vegetable‐genetic‐improvement‐network‐vegin). A recent announcement made by the UK government (Gov UK and DEFRA, 2022) on crop improvement, which is of great importance for the sustainability of UK agriculture, makes it clear that the crops improved by the novel technique of gene editing are soon likely to become an important part of UK Agriculture. As part of a gradual approach towards allowing gene editing as a means of improving yields, stress resistance and resistance to pests and diseases of UK food crops, research into the gene editing of plants in the UK will become much easier. New rules brought forward by the Government in 2022 encourage field trials and other research efforts and a bill introduced in Parliament in May 2022 allows commercial growing of gene‐edited crops in England. Tomatoes that boost the body's vitamin D could be among the first gene‐edited crops allowed on sale in England. One in six people in the UK are deficient in vitamin D, which is vital to strong bones and muscles and helps reduce the risk of cancer. Although the introduction of this new technology, which can also result in increased drought and pest and disease resistance of crops, will be controversial for some and will require an open conversation with the British public to aid public acceptance, it does offer the opportunity of increasing the sustainability, resilience and profitability of UK agriculture.

Conventional plant breeding coupled with the observation that the changing climate in the UK has already had the effect of increasing the UK growing season by up to a month (Carbon Brief, 2016). This means that the period of the year during which many crops can be supplied to the market has been lengthened. Many mature crops can be stored at the end of the growing season, using low‐tech, low‐cost systems before being brought to the market. A growing understanding of postharvest biology now leads growers to use often‐simple techniques to take the heat out of certain crops soon after harvest, thereby prolonging storage opportunities. Advanced packaging and storing methodologies will also extend storage periods by reducing the loss of water from many crops. Such extended storage techniques allow growers the opportunity to benefit from higher prices for products in the ‘off‐season’. In addition to this, many/most higher value crops such as soft fruit, tomatoes, peppers and cucumbers are grown in the UK under more sophisticated protected cropping systems involving glasshouses or polythene tunnels and crop mulches, examples of a system commonly referred to as ‘plasticulture’.

Exploiting modern genetics in combination with modern agroecological crop management techniques to increase productivity and sustainability of UK farms will potentially require more land devoted to crop production. Arable production comprises a relatively small part of the total land area currently required for UK food production (6 million of 17 million ha devoted to agriculture, Benton et al., 2017). Therefore an expansion of this segment of the market should not be an insurmountable problem, particularly as in the future we may reduce the requirement of land for meat and dairy production (Benton et al., 2019). It is often noted that much grazing land in the UK is not of high enough quality for arable farming, but much high‐quality fruit and vegetable production now involve specialist high‐production systems where many crops are no longer grown in soil. Rather, to minimise problems with soil‐borne pests and diseases, artificial growing media are employed which themselves may serve as a support medium for a crop relying on a flowing solution culture for water and nutrient supply. These systems can reduce pollution issues arising from diffuse use of fertilisers and indiscriminate use of pesticides and can also deliver enhanced efficiency of water use (FAO and IWMI, 2017), thereby constituting an important part of the response of the UK farming community to new environmental legislation. Highly‐productive protected cropping systems also do not always require large areas of land, but there are still environmental challenges for these systems as many people already resent the visual impact of such developments in areas of outstanding natural beauty (Rogge et al., 2008). The most sophisticated of these developments, taking profit from modern environmentally‐efficient glasshouse technology, lighting developments, farming systems, etc., may be too expensive for extensive deployment in some parts of the UK market. Nevertheless, novel developments in science and technology still provide lots of opportunities for the industry that will impact positively on UK food and farming and increase the broader social and financial benefits from such sustainable and localised developments. The UK Plant Science Federation (UKPSF, 2019) in its seminal report ‘Growing the Future’ has recognised as a priority ‘Promotion of public‐private partnerships and collaborations with consistent, long‐term R&D policies, and support to bridge the gap between discovery science and commercial application’ and has suggested that future funding should ‘support innovative strategies to develop nontraditional agricultural and horticultural systems’. We propose that intensive production systems of the kind that are currently in use across segments of the UK horticulture industry can be further developed to become more environmentally sustainable and to provide the UK with increased quantities of healthy food to provide increased resilience against environmental and societal changes.

## 4 PLASTICULTURE: A KEY COMPONENT IN ACHIEVING INCREASED FOOD SECURITY AND HEALTHY DIETS

Given the success of much‐protected cropping under plastic in the UK, we consider here the arguments for and against the increased use of plastic in the UK for food production. The use of plastics in crop production systems is a worldwide practice originally introduced in the late 1940s to reduce the cost of agricultural infrastructure (Le Moine and Ferry, 2019). Plastic use in our food system extends beyond crop production, as plastic films are commonly used for the transportation, protection and storage of fresh produce once harvested. Reducing food spoilage/waste is a key part of the food security challenge, and to that end, the industry is working hard to introduce less‐environmentally damaging packaging alternatives.

Plasticulture is proven to boost agricultural productivity in yield‐limiting conditions (Espi et al., 2006; Harrison & Hester, 2018) and is generally considered an inexpensive means to enable the food supply chain to meet food demand and consumer expectations on food quality. Uptake of plasticulture by producers and suppliers in the UK, much like other European countries, is driven by what can be almost immediate benefits of the practice. Current use in the UK is driven by the competitive advantage of producing early‐season marketable yields (DEFRA, 2011). Plasticulture in the UK is also used to increase yields at other times in the season, reduce agrochemical loads and provide protection from birds, pests, frost and other adverse weather conditions. Following crop diversification programmes in Europe during the 1970s, plasticulture evolved to incorporate a range of polymers (Orzolek, 2017). Development of new polymers has facilitated new growing techniques such as soil‐less cultures; hydroponics, aeroponics, vertical farming and controlled‐environment agriculture more generally (Rubio‐Asensio et al., 2020). Within the UK, 52% of all soft fruit is grown under protection; 6222 hectares of 11,966 hectares are destined for crop production (Ridley et al., 2020). However, this is a generalisation of the sector and does not accurately represent the ratio of protected to unprotected cropping on a crop‐by‐crop basis. 92% of UK strawberries, for example, are now grown under polytunnels, often on raised platforms in plastic bags, pots or troughs to maximise yield, quality and ease of harvest (Ridley et al., 2020). Recently‐developed polymers that alter the spectral quality of solar radiation may be used to manipulate plant morphogenesis, improving the quality, aesthetics and taste of the produce (Orzolek, 2017). Crop protection from pests and diseases is often aided by the introduction of plasticulture. Considering the increase in domestic policies focussing on landscape recovery, competition for land use between food production, restoration and climate mitigation projects will intensify. New growing techniques such as those outlined above may help minimise the area devoted to crop production in the UK without compromising the productivity, thereby boosting domestic production of accessible, affordable, high‐quality fruit and vegetables whilst freeing up land for landscape recovery.

The effectiveness of plastic applications is dependent upon the environmental conditions, crop type, location, season and quality and spectral properties of the film (Gao et al., 2019; Orzolek, 2017; Steinmetz et al., 2016). Because of variation in the type and magnitude of environmental stresses that affect crop yield and water‐use efficiency on a local to the international scale, using plastic applications to boost crop production does not always equate to a more profitable production system because of the upfront costs and the effectiveness of the plastic application itself. For example, plastic mulch applied to wheat fields in China during the crop establishment phase was only cost‐effective where water availability was low. When the soil water content exceeded 60%, nonmulched plots showed better cost–benefit ratios and bigger net annual incomes (Xie et al., 2005).

A ‘best estimate’ of the extent of plasticulture in the UK, provided by Scarascia‐Mugnozza et al., 2011, indicates that at that time, the UK grew around 12,000 hectares of crops under cover, 10,000 hectares of which were mulch films. There were estimated to be 2500 hectares of greenhouses and polytunnels and 1400 hectares of low tunnels. Data compiled by DEFRA suggest that an area of 7986 hectares was covered by plastic in 2017. Due to the inconsistencies in estimates, determining the trends of plastic‐protected cropping over recent years proves difficult. Estimates suggest that the use and area covered by plastic applications in the UK have generally increased and continue to do so, subject to annual fluctuations (DEFRA, 2011; Steinmetz et al., 2016). Ridley et al. (2021) have suggested that between 2011 and 2017 the area of edible protected crops in the UK increased by 13.5%. Between 2017 and 2019 the area of edible protected crops decreased by 30%, but the crop value increased by 9.6% over the same period (DEFRA, 2021; Ridley et al., 2021). Some of the increase in crop value is due to a general increase in crop quality arising from both agronomical and genetic developments. More value is added by the development of sophisticated glasshouse systems extending the cropping season at both ends of the year, from the period when cropping is possible under plasticulture. This development and the use of protected cropping generally may be driven by consumer expectations of the ready availability of seasonal crops in all months of the year and under potentially more challenging growing conditions as our climate changes (Else & Atkinson, 2010). In the UK, the economic advantage of growing under polytunnels has been most effective for soft fruit growers, particularly strawberry growers (Lewers et al., 2017; Warner et al., 2010). An increase or decrease in the area covered by plastic is not necessarily reflective of a proportional change in the volume of plastic used. Modern cropping techniques may use multiple plastic applications such as fleece wrapping of young strawberry plants inside a polytunnel late in the winter and early in the spring.

Across the world, an increase in water‐use efficiency is one of the main incentives for farmers to use more plastic and as the climate emergency grows, this is very likely to increase in importance as a major driver of innovation in the UK. Plastic mulch films and fleeces, commonly made of low‐density polyethylene (LDPE) or polypropylene (PP), are durable, flexible and cost‐effective. Both applications conserve soil moisture by reducing surface evaporation. Higher soil water contents will aid seed germination of crops sown under a mulch film or fleece. It is anticipated that expenditure on irrigation and water management will be reduced once the plastic applications are integrated into the crop production system (Chang et al., 2013; Ruíz‐Machuca et al., 2015; Steinmetz et al., 2016; Wang et al., 2016). The economic effect of using plastic mulch films may translate to an approximate 25% saving in water costs (Steinmetz et al., 2016), particularly when the existing crop production system operates in arid regions with low‐water availability (Biswas et al., 2015). In China, plastic film mulch decreased evapotranspiration from a maize crop (Fan et al., 2017). Importantly, because of changes in crop energy balance, the plastic film mulch accelerated plant growth and advanced maize maturity, and thus improved biomass production, grain yield and water‐use efficiency. Water availability for irrigation is a growing issue around the world and this is increasingly the case, particularly in the south of the UK (Rio et al., 2018). Water savings combined with increases in yield can be of great importance to farmers as the climate changes.

Plastic mulch films, fleece, polytunnels and greenhouses induce localised soil warming. These plastic applications trigger a greenhouse effect as condensed water trapped beneath the film absorbs the IR radiation reflected by the soil. Variations in the colour of the film translate to a different soil warming effect as different films allow different parts of the incident solar spectrum to penetrate the film. Transparent films generally provide the largest soil warming effect by transmitting between 85 and 95% of the solar spectrum and trapping long‐wave infrared radiation (LWIR) (Snyder et al., 2015; Steinmetz et al., 2016), increasing soil temperatures, in some cases by more than 7°C (Wang et al., 2016).

The potential benefits of early‐season soil warming are shown by the work of Gregory and Marshall (2012), where the analysis of historic climate warming in Scotland shows very significant yield benefits as a result of early chitting of seed potatoes in response to soil warming of only around one‐degree centigrade. An increase in soil temperature resulting from the use of plastic films covering the soil induces early tuber growth and seed germination of other crops, which extends the growing season of many crops and increases the choices of crops that may be grown in particular regions (Chang et al., 2013; Gosling et al., 2014). Within the UK, plastic mulch films, fleece, polytunnels and greenhouses provide protection against frost to produce an early‐season yield, when the price and demand of domestic production are high. Many economically valuable crops, particularly soft fruits, require warmer temperatures than those occurring in the UK in all but the mid‐summer months and so the use of plastic will invariably increase productivity in spring and autumn (Lewers et al., 2017).

Plastic mulch films and fleeces can prevent the spread of viral diseases by repelling insects (Olle & Bender, 2010; Victor & Julius, 2018). Additionally, both applications may attract beneficial predators such as beetles and spiders, which limit aphid‐borne diseases. Plastic mulch films are also effective in reducing weeds, bacteria, fungi and viruses through soil solarisation and can be used in conjunction with other soil sterilisation techniques. However, the new microclimatic conditions under these plastic applications may exacerbate the incidence of diseases such as powdery mildew, caused by the fungi *Podospaera xanthii* (British Summer Fruits, 2017). Generally, using plastic reduces the reliance on pesticides and herbicides and allows the use of integrated pest management (IPM), reducing the cost of pest and disease control per unit of crop production (Chang et al., 2013; Qi et al., 2020). Although the use of plastic mulch films and fleeces may reduce the reliance on fungicides compared with organic mulches, plastic mulch films and fleeces may create a favourable environment for fungal development (*Botrytis cinerea)* compared with uncovered cropping systems (DEFRA, 2011; Meyer et al., 2021).

Plastic structures, particularly greenhouses, polytunnels and nets provide protection from adverse weather conditions such as heavy rainfall or hail, high winds, extreme heat and exposure to intense sunlight—all of which may damage a crop (Orzolek, 2017). Cost/benefit ratios will determine the choice of protective covering (Ahamed et al., 2019). In Northern Europe, both glass greenhouses and polytunnels are used for extended production of a range of higher value crops throughout the year. The increased availability of these high‐value, high‐quality crops may give consumers an incentive to consume more healthy food. Greenhouses in Mediterranean countries are mainly plastic‐covered (Chang et al., 2013; Orzolek, 2017). Even in northern Europe, plasticulture continues to be popular and between 1980 and 2015, the consumption of agriplastics in Germany rose from 136,000 tonnes to 635,000 tonnes (Brandes et al., 2021). Each covering material has pros and cons related to the cost and longevity of the structure, whether the material is compatible with particular growing and climate‐control systems, the effect of the material upon the plant microclimate, the external climate and any maintenance costs. Direct natural light may scorch the uppermost leaves of the crop and cast a shadow on adjacent crops within a glass greenhouse and some expenditure on shading is therefore required. Due to the radiative properties of plastic films, incoming light is scattered to reduce the risk of scorching and increase the proportion of the canopy receiving light, due to better light distribution (Espi et al., 2006). Additives such as Lumisol may be used to positively manipulate the radiative properties of the plastic film. Lumisol minimises the condensation of water on the plastic film, which maximises light transmission and reduces the risk of fungal diseases such as *Botrytis cinerea*. The low cost of plastic has resulted in plastic greenhouses becoming a cost‐effective but highly‐productive method of growing in many parts of the world (Orzolek, 2017).

The recoverability, reusability and recyclability of plastics within greenhouses are better than open‐field systems that use mulches, fleece and nets, as degradation rates are lower, reducing plastic pollution and the loading of microplastics into agricultural soils (Hablot et al., 2014). Degradation rates in open‐field systems are higher due to greater exposure to sunlight, extreme weather and physical fragmentation from fauna. Glass greenhouses support many sophisticated growing techniques such as vertical farming, but variations of this development are also used in polytunnels often employing soil‐less substrates as a rooting medium. Soil‐less growing presents little threat to soil degradation and a reduction in soil quality via plastic pollution, loss of organic carbon, compaction or erosion (Manos & Xydis, 2019; Sambo et al., 2019), issues that already cost £1.2 billion a year in England and Wales (Environment Agency, 2019). However, whether glass or plastic is used to create the greenhouse, both growing methods are plastic‐intensive so should be considered as a form of plasticulture.

## 5 THE POSITIVE IMPACTS OF THE INCREASING USE OF PLASTICULTURE IN UK HORTICULTURAL PRODUCTION

**FIGURE 3:**
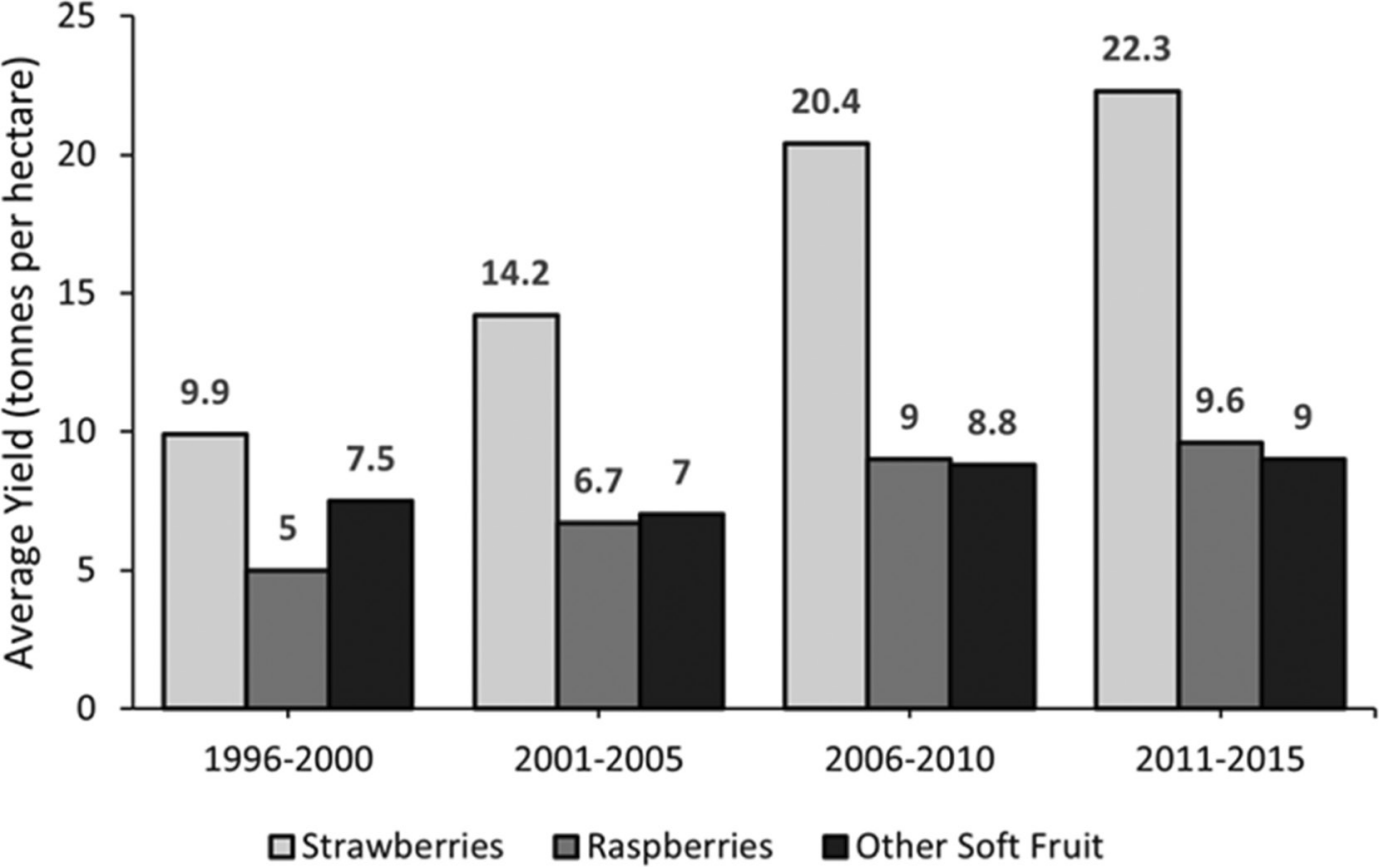
Cusworth et al. A graph illustrating the average yield (in tonnes per hectare) of strawberries, raspberries and other soft fruit in the UK between 1996 and 2015, in 5‐year periods. Data collected by British Summer Fruits (2017)

While the success of UK agriculture and horticulture in recent years is not entirely due to research, development and deployment of agriplastics, some crop production systems have been revolutionised by different applications of plastic. In the UK, the average yield of raspberries increased between 1996 and 2015 from 5 tonnes per hectare to 9.6 tonnes per hectare (see Figure 3), coinciding with an increase in the area of raspberries grown under polytunnels from 25 hectares to 1304 hectares (British Summer Fruits, 2017; Garthwaite et al., 2017). Hanson et al. (2011) have reported that raspberries grown under polytunnels are 12% less likely to develop a fungal disease (*Botrytis cinerea*) than crops grown in the open field. Typical diseases such as spur light (*Didymella applanate*) and cane anthracnose (*Elsinoe veneta*), were significantly reduced under polytunnels (Demchak, 2009). Plasticulture provides near‐year‐round supply and better quality of raspberries, whilst reducing wastage due to a decrease in spoilage and low‐grade fruit. This is evident as UK‐produced raspberries marketed for the year increased by 9900 tonnes between 2002 and 2015 (British Summer Fruits, 2017).

The strawberry is one of the most economically important fruit crops worldwide (García‐Tejero et al., 2018; Lewers et al., 2017). Approximately 3900 hectares of strawberries in the UK are either grown under polytunnels, mulches or fleece, which resulted in an increase in the average yield of 9.9 tonnes per hectare to 22.3 tonnes per hectare from 1996 to 2015 (British Summer Fruits, 2017; DEFRA, 2011). Plastic coverings in the soft fruit industry are rarely used in isolation, more often in conjunction with other plastic applications. For example, strawberries may be grown in a soil‐less substrate on a raised platform (allowing easier harvesting) under a polytunnel with a fleece wrapping during the coldest months. These new growing techniques have provided effective protection against fungal diseases, particularly *Botrytis cinerea* (Evans, 2013; Lewers et al., 2017). This once‐prevalent disease limited the yield of strawberries and other soft fruits to around 50% Class 1 yield, due to a significant reduction in shelf life and the presence of rot, which has since increased to 90% following the use of plastic applications (British Summer Fruits, 2017; Evans, 2013). As more produce meets the criteria of supermarkets and groceries, food waste on the farm is greatly reduced. The benefits provided by plastic covers have resulted in the strawberry crop becoming one of the most economically important crops within the UK. Domestic production of strawberries was valued at £429 million in 2020, an increase in 165% since 2016 (DEFRA, 2021). Although berries do not currently represent a significant portion of the typical UK diet, the increased production of these crops may positively impact consumer health, food availability and wider food security issues. Increasing the production of off‐season produce could fill an otherwise seasonal void in the consumption of specific crops and may reduce the price of off‐season produce. An increased availability and accessibility of healthy, high‐quality products allow consumers to form consumption habits and adopt healthier diets (Kuchler & Arnade, 2016).

In another example, the use of plastic in blackcurrant cultivation is limited, but important nonetheless. The soil beneath blackcurrant bushes can be covered in polyethylene mulch, with the primary aim of suppressing weeds. Mulch film intercepts a proportion of rainfall, reducing the amount of water made available to the crop, which necessitates the use of an irrigation system. Cherries are often grown under multiple plastic structures, consisting of nets and polytunnels. These covers are used on approximately 80% of the cherry crop in the UK (DEFRA, 2011), which is a relatively recent innovation that, along with low‐cost storage techniques, has transformed the industry. These applications provide protection from intense precipitation events and prolonged periods of solar irradiation, which may cause fruit splitting, which has historically been a major problem for the UK industry (Mika et al., 2019).

Plastic mulches, fleeces and nets are also used in the UK for the cultivation of vegetable brassicas, leafy vegetables, fruiting vegetables and root crops. There is a lack of available data about the measurable effect of plastic mulches, fleeces and nets on crop yield and other parameters in the UK. One study in 1988 noted a 14.7% difference in carrot yield under plastic mulches compared with uncovered crops (Peacock, 1991). The potential advantages of using plastic applications in UK horticulture are, however, widely recognised. Plastic mesh and nets are extensively used for swede and turnip cultivation. These applications are laid in the early spring, pre‐ or postsowing or planting to increase the soil temperature and provide protection against frost. This is temporarily removed and re‐laid periodically to monitor the crop and apply field amendments. Once the crop is established, the films are removed. Due to a ban on some pesticides that control cabbage fruit fly (*Delia radicum*), the use of nets on brassica and root crops has increased to prevent fly damage. Approximately 95% of turnips and swedes grown in the UK are covered by nets for pest protection (DEFRA, 2011). Although plastic nets generally reduce disease, poor air circulation under the nets increases the risk of *Alternaria* (Saharan et al., 2019). Within the UK, plastic covers in potato production are primarily used to induce soil warming with the aim of producing earlier and higher yields. Plastic use in UK potato production has reduced due to developments in storage facilities and pressure from cheaper international imports, but plastics may be used more in the future to conserve soil moisture to control potato scab (*Streptomyces scabies*) and improve water‐use efficiency following stricter water‐use regulations and developing climate change (Biesbroek et al., 2010; Daccache et al., 2015).

## 6 PROBLEMS WITH PLASTICULTURE

While the UK horticultural industry has been extremely successful in recent years, the environmental cost of plastic production and plastic pollution is regarded as a generational challenge that faces the earth system complex and any evolving crop production system should not further complicate this problem. The effects of plastic pollution are global and the distribution of plastic waste is ubiquitous (Guo et al., 2020; Scheurer & Bigalke, 2018; Yates et al., 2021). Plastic pollution is a complex issue occurring on a scale of nanometres to metres. Most plastic is persistent and durable. Environmental effects range from physical blockages in rivers to cytotoxicological effects like inhibited spermatogenesis in earthworms (Kwak & An, 2021). In an important recent paper, Persson et al. (2022) reviewed the scientific literature relevant to quantifying the planetary boundary for novel entities and they highlight plastic pollution as a particular aspect of high concern. An impact pathway from the production of novel entities to impacts on Earth system processes is presented. This can be a key development in the assessment of plastic pollution loads and effects. The planetary boundaries approach has been used to great effect as we have sought to quantify the global impacts of the way that we lead our lives (Rockström et al., 2009).

Most plastics are derived from fossil‐fuel feedstocks and therefore synthesis, use and disposal can have a big effect on global GHG emissions. Assuming that agricultural plastics comprise 2–3.5% of annual global plastic production, the ‘cradle‐to‐resin’ emissions of production total 17–30 million tonnes of carbon dioxide equivalent (Mt CO₂ eq), predicted to increase to 27–47 Mt CO₂ eq by the end of 2030 (FAO et al., FAO, 2021; Hamilton et al., 2019). Emissions from European agriplastic production total 1.5–2.1 Mt CO₂ eq based on current estimates. ‘Cradle‐to‐resin’ estimates omit GHG emissions from the conversion stage; processing polymers into final products, and the end‐of‐life stage; the treatment and disposal of plastic waste, which is thought to contribute an additional 14–24 Mt CO₂ eq to current estimates (Zheng & Suh, 2019). In comparison to the 8.26 Gt CO₂ eq emitted from global transportation, ‘cradle‐to‐grave’ GHG emissions from plastic production are minor (WRI, CAIT, 2021). Nevertheless, all of this undermines the efforts by the food industry to address the Climate Emergency declared in the UK, the Paris agreement and subsequent Conference of the Parties (COP), which aims to limit global warming to 1.5°C (United Nations/Framework Convention on Climate Change, 2015). Although research is in its infancy, the degradation of plastics in the environment may increase GHG emissions (Shen et al., 2020), with the scope to affect biogeochemical cycles in the soils, and consequently the GHG flux and global warming potential (GWP) (Ren et al., 2020). Once agriplastics reach their end of life, they are often left on the ground, ploughed into the soil, burned or disposed of in landfill. Between 16 and 50% of agricultural plastic waste (APW) is not managed (FAO, 2021). The estimate is region‐specific, difficult to quantify and is considered an underestimate as many farmers store unknown quantities of APW on the farm (Blanco et al., 2018). The impact of mismanaged APW on climate change is relatively unknown.

The management of much APW continues to be inappropriate (Briassoulis et al., 2013). Recycling polytunnel and greenhouse films is more achievable due to low levels of contamination. Plastic mulch films, fleeces and nets removed from fields are often contaminated with soil, pesticides and fertiliser throughout their application, so are often rejected by recycling facilities (Aznar‐Sánchez et al., 2020). If recycling facilities accept the contaminated film, growers are charged per unit of weight, which is costly and inefficient (APE, 2019). Following the implementation of the ‘National Sword’ policy, APW exports from the EU were banned from entering China for recycling, resulting in a larger proportion of APW being diverted to landfill (APE, 2019). Due to the high cost of sending APW to landfill, the traditional method of disposal has been to burn films on the farm (Sintim & Flury, 2017). Each of these approaches raises environmental concerns. Burning APW poses a threat to air quality and consequently public health, whilst APW disposed of in landfill can result in the leaching of a range of chemicals into the broader terrestrial environment and also contaminate the marine environment (Brodhagen et al., 2017).

In the absence of a regulated APW management system, farmers are left with limited choices for disposal. As a result, many growers store APW in temporary farming areas, with little knowledge of what to do with the material (Blanco et al., 2018). However, the growth of national collection schemes (NCS) and commercial operations dedicated to extending producer‐responsibility of agriplastics in Europe is promising. APE UK, a NCS founded in 2020, expects to collect 22,500 tonnes of APW by 2022, a significant improvement from 2800 tonnes in 2021. Although there are challenges with recycling infrastructure and the low value of recycled plastic, the future of APW management appears promising.

Although recycling and reuse of agriplastics are practiced, it is difficult to recover plastic from applications where it begins to degrade shortly after use, this is particularly the case for plastic mulch films net and fleeces. The thickness of mulch films influences the recovery rate. Thicker films inherently use more plastic per unit area but have high rates of recovery. Thinner films are more likely to have low rates of retrievability due to lower structural integrity (Liu et al., 2014). Combined with the accelerated disintegration from extreme weather events and physical damage from fauna, some films are extremely difficult to recover (Hablot et al., 2014). Therefore, many plastic films are often left in the field to disintegrate further, regardless of whether this act of disposal is intentional or unintentional (Moreno et al., 2014).

The primary receptor of agriplastic pollution is agricultural soil. The effect of agriplastic pollution here is compounded by other agricultural inputs such as slurry, biosolids, municipal waste, plastic‐coated fertiliser, agricultural machinery, atmospheric and fluvial deposition. It is unsurprising therefore that agricultural soils are likely to contain among the highest concentrations of microplastics worldwide (Nizzetto et al., 2016). These soils are also the most understudied. In addition, there is no unified procedure for the processes of extraction, impurity removal and identification of microplastics or nanoplastics, which makes quantifying and identifying point sources of plastic pollution in agricultural soils difficult (Qi et al., 2020).

Studies of plastic pollution in agricultural soils across the world vary with most focussing on microplastic contamination. In extreme cases where mulch film was buried or ground into the soil for over ten years, residual levels have varied between 50 and 260 kg per hectare and have exceeded 380 kg per hectare in other studies (Changrong et al., 2014; Liu et al., 2014; Qi et al., 2020). 63,000–430,000 tonnes of microplastics a year are thought to be applied to European farmlands through biosolids and fertiliser application (Nizzetto et al., 2016). Microplastic contamination in biosolids is thought to range between 10^3^ and 10^4^ particles per kilogram (Kumar et al., 2020; Qi et al., 2020). Due to low rates of plastic degradation in soil, the consistent application of plastic agricultural films and field amendments are thought to compound the concentration of microplastics in agricultural soils. Currently, there is no published systematic research that identifies the microplastic concentrations of agricultural soils in the UK. The effects of plastic pollution on the terrestrial environment, particularly agricultural soils are also understudied (see Table 1) (Qi et al., 2020; Zhang et al., 2020).

**TABLE 1:** A review of the existing global studies that have determined microplastic concentrations in agricultural soils across the world. Data collected from Büks and Kaupenjohann (2020).

Region	Number of studies	Median microplastic concentration (particles kg^−1^)	Range of microplastic concentration (particles kg^−1^)
East Asia	9	1112.5	0–690,000
Europe	3	2830	0–528,000
Middle East	1	333.5	0–1133
The Americas	4	1200	0–10,200

During the production of plastics, various additives and plasticizers are included to tailor the polymer to a specific application. Phthalates, for example, are plasticizers that are used to increase the plasticity of polymers used in agriculture. Shortly after exposure, these chemicals may be released into the environment as they are loosely incorporated into the polymer (Steinmetz et al., 2016). Examples include diethylhexyl phthalate (DEHP), polychlorinated biphenyl (PCB) and decabromodiphenyl ether (DBDE), most of which are endocrine disruptors and may impair human health in high concentrations (Harrison & Hester, 2018; de Souza Machado et al., 2018). Once plastics decay into the smaller microplastic and nanoplastic particles, the broken carbon backbone of the polymer may be introduced to new chemical groups (Gewert et al., 2015). Toxicants may readily sorb to these particles, resulting in a host of toxicological effects (Velzeboer et al., 2014).

It is very difficult to quantify the impact of microplastics, nanoplastics and phthalates from agriplastics on human health, due to the difficulty in point‐source tracing and numerous background pathways of contamination. Humans are exposed to plastic pollution through common pathways of ingestion, inhalation and cutaneous contact. Food, amongst other things we ingest, may be a source of contamination. In Catania, counts of plastic particles <10um found in fresh fruit and vegetables ranged from 87,600–124,900 per gram of produce (Conti et al., 2020). It is thought that humans may ingest over 100,000 plastic particles a day, consuming the weight of a credit card in plastic a year (Koelmans et al., 2020).

Due to the size of microplastics and nanoplastics, they are highly mobile, able to pass through cell membranes within the body and accumulate in human tissue (Prata et al., 2020) and plastic particles have been found in the human placenta, gut tissue and faeces (Ragusa et al., 2021; Schwabl et al., 2019). The proposed health effects of these particles and chemicals are overwhelmingly negative: inhibition of metabolic function and homeostasis (Deng et al., 2017; Prata et al., 2020), inflammation triggering an immune response or oxidative stress (Deng et al., 2017), respiratory conditions e.g. ‘flock worker's lung’ (SAPEA, Science Advice for Policy by European Academies, 2019), increased severity of autoinflammatory and autoimmune conditions (Yan et al., 2021), and cytotoxicity and intracellular damage (Danopoulos et al., 2021; Prata, 2018).

In highly contaminated agricultural soils, macroplastics will inhibit nutrient and water transport within the soil (Liu et al., 2014). The presence of macroplastics changes the physical structure of the soil, limiting plant uptake of moisture and nutrients, the development of an established root network and seed germination. Broad decreases in these variables can result in a reduction in crop yield. In Xinjiang, where soil mulches are commonly used to reduce soil water loss, when macroplastic pollution exceeded 200 kg per hectare, a 15% decrease in cotton production was observed (Liu et al., 2014; Zhang et al., 2020). Most studies show a negative effect of soil microplastics on plant physiology (Boots et al., 2019; Bosker et al., 2019), but the presence of polyester fibres in soil has been shown to increase root biomass (de Souza Machado et al., 2018). This suggests that polymers may induce different responses according to shape and type (Boots et al., 2019). The mechanisms behind these effects are currently unknown. It is thought that nanoplastics may accumulate in plant tissue through submicrometre openings in roots and may then be translocated through vascular networks (Lin et al., 2020). Research on plastic pollution and crop health is in its infancy. Current studies often test the effects of plastic pollution at unrepresentative environmental concentrations and lack data on how the presence of microplastics affects long‐term crop health, particularly nutritional characteristics.

The structure, microbiology and the physical and chemical properties of soil are thought to change under varying levels of plastic pollution. Changes in soil structure are more likely to become prevalent in the presence of macroplastics (Qi et al., 2020). Macroplastics form an impermeable physical barrier, reducing aeration that may trigger soil anoxia, which is a serious issue for all crop plants (Steinmetz et al., 2016). Significant changes in soil pH have been noted in the presence of microplastics. It is thought that microplastics may impact the cation exchange in the soil and result in both positive and negative changes in soil pH (Boots et al., 2019; Zhao et al., 2021). Changes in soil chemistry have a direct effect on soil microbiology. The activity of soil micro‐organisms directly impacts soil nutrient cycling, which is important for plant growth. Microplastics may directly affect the activity of soil micro‐organisms, which can influence the decomposition of soil organic matter and carbon sequestration, both positively and negatively (Xiao et al., 2021). The effects on soil micro‐organisms are exclusive to the shape, type, concentration and exposure time of the microplastic and are magnified higher up the soil food web (Lin et al., 2020; Zhao et al., 2021).

Profound effects of microplastics have been observed on earthworm populations. Earthworms influence soil formation, maintenance, structure and fertility (Edwards, 2004). Earthworms and other soil biota may misidentify plastic particles as a food source. As these particles have no nutritional value or serve any function, energy allocation for growth and other metabolic processes decreases (SAPEA, Science Advice for Policy by European Academies, 2019). Depending on the severity of pollution, the ingestion of microplastics may increase the mortality rate of earthworms as a result of significantly reduced feeding (Huerta Lwanga et al., 2016). A decrease in feeding activity not only affects the earthworm itself but reduces the mixing of soil by living organisms, a key process to maintain healthy soils through effects on soil water balance and nutrient cycling (Blouin et al., 2013). Bioturbation may also exacerbate plastic pollution. Earthworms are capable of transporting microplastics through the soil profile, exposing other soil biota and increasing the residence times of these particles (Rillig et al., 2017). Plastic particles do not readily degrade within the soil and may remain within the soil profile for decades.

The societal impact of the operation of our food system is often overlooked (Yates et al., 2021) and this is particularly the case with the use of plastics in rural locations. Conflicts have developed between nonfarming residents in rural communities and farmers. Plastic structures can be displeasing to some and visible in valued landscapes. Due to the large scale of some of these operations, the extension of the picking season can be very disturbing to those living nearby. Many have voiced complaints about the noise, traffic and dust from greenhouses and polytunnels (Evans, 2013). These negative effects should be contextualised and balanced with counter‐benefits such as increased employment opportunities, the provision of foods that are important for a healthy diet (Lillywhite, 2014) and a significant limitation of chemical pollution and GHG emissions from tilling of soil, etc. Particularly intensive operations can be located on brownfield, formally industrial sites in peri‐urban locations.

## 7 THE WAY AHEAD

Following Brexit, many UK trade policies are changing with restrictions on some imports and relaxation of standards and tariffs on other products. Restructuring of Britain's farming and land‐use policies will provide an opportunity to address some of the practices leading to high emissions of greenhouse gases from the import of food and from farming practices that have become integral to the intensive agriculture practiced in much of the British Isles (Godfray et al., 2010). All of this and the development of the global Covid pandemic and the conflict in Ukraine have led to a renewed discussion with the public about the nature of the UK food system including a possibility for more self‐sufficiency and resilience in food production for the UK market.

The EAT Lancet Commission (Willett et al., 2019) and others (Global Food Security, 2021) have emphasised the need for the adoption of a diet for both the health of the individual and the health of the planet. For many in the UK, this can involve, for example, eating reduced amounts of red meat and such a dietary shift could free up a considerable amount of grazing land in the UK for the aforementioned land‐sparing strategy (with its predicted positive impact on biodiversity). A healthier diet for most people means the consumption of considerably more fruit and vegetables. These foods should be made more affordable to many. Much of the considerable area of land currently used for grazing in the UK (Benton et al., 2017) commonly have soil, which is of a quality that is unsuitable for much agronomical production. If we assume that much of existing agronomy can be sustained on what is a comparatively small proportion of the UK's agricultural estate then much additional food needs to be produced in specialised units developed on sites previously considered unsuitable for crop production (Walsh et al., 2022). It is partly for this reason that we propose here that protected cropping production systems for high‐quality fruit and vegetables could allow intensive production on comparatively small areas of land. High productivity can be achieved in controlled environments, potentially involving some vertical farming with artificial rooting substrates or solution culture. Such systems can be operated with a very limited environmental impact. Our expectation is that these facilities can be used to grow increased quantities and an increased range of crops in the UK, both by extending the production season of many crops and by enhancing the productivity of a range of crops during the main UK growing season, thereby future‐proofing the availability of healthy affordable food in the UK. This system can be used to encourage new local business development, the development of circular agriculture and greater economic resilience (see Figure 1).

While there are many benefits from protected cropping, there are many issues with the widespread use of plastics. In particular, there needs to be multi‐disciplinary action to remediate the issues of agriplastic pollution and waste, both in novel and existing operations. Interventions must be holistic and take a ‘cradle to grave’ approach (Zheng & Suh, 2019). The environmental effects of agriplastics can be mitigated by following the principles of source‐pathway‐receptor‐consequence (SPRC) and 6‐R's; refuse, redesign, reduce, reuse, recycle and recover (FAO, 2021).

Our consideration of the pros and cons of plasticulture is not a consideration of whether or not to decrease plastic use. Rather we believe that plastic use currently plays many essential roles in UK food and farming. We must consider how to develop the agriplastics industry into a sustainable, circular economy (Ellen Macarthur Foundation, 2021). Strategies to reduce the global carbon footprint of plastics include increased use of bio‐based feedstocks and decarbonising the plastic supply chain by using renewable energy for the production and processing of plastic waste (Zheng & Suh, 2019). APE Europe has published criteria for farmers to enable them to practice ‘plastic‐neutral farming’. For plastics on the farm to have a greatly reduced effect on the environment, farmers are encouraged to work to criteria that ensure that plastic for a range of applications: is recyclable and eligible for collection, has minimal visual environmental impact and causes minimal soil contamination because of improved recoverability (to optimise waste collection). Farmers are also urged to collaborate with collection schemes (APE, 2019). Facilities of the kind proposed here can be located in less highly valued locations in peri‐urban and even in urban environments close to markets, thereby reducing the impacts of transport for just‐in‐time delivery.

While scenario planning has highlighted some advantages of a localised food system it is clear that because we are a small, highly‐populated country we cannot become completely self‐sufficient in food. A change in our everyday diet may help in terms of increasing self‐sufficiency, but there are many fruits and vegetables, which should be part of a healthy diet but that cannot be grown year‐round in the UK, even with protected cropping. A large proportion of fresh fruit and vegetables imported into the UK is sourced from growers in southern Spain, a comparatively short supply chain (Garnett et al., 2020), but year‐round fruit supply to the UK requires access to production in most parts of the world. A less diverse supply chain is not necessarily a more resilient supply chain, a vulnerability that has been recently exposed by Brexit, COVID‐19 and the Ukraine conflict. If particular regions of the world experience political instability, social disorder or a natural disaster that destabilises their own national food security, protectionism may divert produce away from the UK to local populations for a prolonged length of time. Considering that the UK food system functions on a just‐in‐time basis, the availability of fresh produce to the consumer could diminish in only a few days in response to a quite localised event many hundreds or thousands of miles from the UK. A good example of this was the eruption of an Icelandic volcano in 2010, which limited the supply of Kenyan fruit to the UK for several weeks (Justus, 2015).

While we argue here more UK production of healthy food will enhance the resilience of our food system, diversity in sources of food for the UK will inevitably be needed in the future.

Relatively energy‐efficient overseas supply chains are required for us to decrease the environmental impact of our diet. We have given examples of production in parts of the world where such supply chains exist and where high radiation levels can mean the high productivity of many crops. Trading relationships of this kind and what will be a re‐invigoration of our trading relations with the EU will be key to the sustainability of our food system. Enhanced, domestic production and enhanced local production combined with smart trading relations will also combine to reduce food waste in the food chain. An increased emphasis on local food production should be used to educate consumers about healthy food and how they can act to reduce domestic food waste.

In order to develop a more secure, food system that increases the accessibility of foods and ensures the accessibility of healthy diets for increased numbers of people, some increase in domestic production seems desirable. In line with the NFU's slogan to ‘Back British Farming’ (NFU, 2015), expanding the area of protected cropping in the UK through plastic use could provide cost‐effective benefits for the nation through an increase in agricultural productivity. Further health‐driven dietary changes for our population could, at the same time, provide substantial amounts of land for environmental restoration. Such developments where many new production systems could be in peri‐urban/urban locations, coupled with a land‐use strategy to encourage biodiversity, will help our population develop a more general appreciation of land management strategies and farming and food production and an understanding of the health benefits of particular diets whilst delivering a healthier environment for all of us. A reduced requirement for transport will enhance food system sustainability. Much of the science for these proposed developments are already available to us. New controlled‐environment technology coupled with the UK Government's apparent backing for the adoption of gene editing in the development of UK crops is substantial opportunities, which will allow us to address one of the UK's key challenges for the future—how to feed its population more adequately and reliably while also improving environmental and social sustainability.

## 9 CONFLICT OF INTEREST

The authors declare that the research was conducted without any commercial or financial influence that could be interpreted as a potential conflict of interest.

10

## Data Availability

Data sharing not applicable ‐ no new data generated, or the article describes entirely theoretical research
